# Pathogenic Bacterial Detection Using Vertical-Capacitance Sensor Array Immobilized with the Antimicrobial Peptide Melittin

**DOI:** 10.3390/s25010012

**Published:** 2024-12-24

**Authors:** Sun-Mi Lee, Jun-Ho Song, Kyo-Seok Lee, Kyung-Hwa Yoo

**Affiliations:** 1 Department of Physics, Yonsei University, Seoul 03722, Republic of Korea; sk2sjy@naver.com (J.-H.S.);; 2 Nanomedical Graduate Program, Yonsei University, Seoul 03722, Republic of Korea; 3 Division of Rheumatology, Department of Internal Medicine, Yonsei University College of Medicine, Seoul 03722, Republic of Korea

**Keywords:** antimicrobial peptides, melittin, clinical bacteria detection, vertical-capacitance sensor, anti-biofilm formation

## Abstract

The rapid and reliable detection of pathogenic bacteria remains a significant challenge in clinical microbiology. Consequently, the demand for simple and rapid techniques, such as antimicrobial peptide (AMP)-based sensors, has recently increased as an alternative to traditional methods. Melittin, a broad-spectrum AMP, rapidly associates with the cell membranes of various gram-positive and gram-negative bacteria. It also inhibits bacterial biofilm formation in blood culture media. In our study, bacterial growth was measured using electrical vertical-capacitance sensors with interdigitated electrodes functionalized with melittin, a widely studied AMP. The melittin-immobilized vertical-capacitance sensors demonstrated real-time detection of both standard and clinically isolated bacteria in media. Furthermore, these sensors successfully detected clinically isolated bacteria in blood culture media while inhibiting bacterial biofilm formation. Melittin-immobilized vertical-capacitance sensors provide a rapid and sensitive pathogen detection platform, with significant potential for improving patient care.

## 1. Introduction

Pathogenic bacterial infections are associated with various issues that affect human quality of life and cause serious diseases such as sepsis [1]. Traditional methods for detecting these pathogenic bacteria rely on the analysis of colony-forming units after bacterial culture and nucleic acid detection using polymerase chain reaction (PCR) and mass spectrometry. However, these techniques are often labor-intensive, time-consuming, and challenging to integrate into comprehensive evaluation methods. To address these limitations, various biosensors with enhanced specificity and sensitivity have been developed for the detection and identification of pathogenic bacteria [2]. The principle of biosensors involves converting the interactions between pathogenic bacteria and corresponding biorecognition elements such as antibodies [3], aptamers [4], clustered regularly interspaced short palindromic repeats (CRISPR)/CRISPR-associated nucleases [5], and antimicrobial peptides (AMPs) [6] into electrochemical signals (e.g., impedance, capacitance) [7,8] or optical signals (e.g., fluorescence, surface-enhanced Raman scattering) [9,10]. Antibody- or aptamer-based detection biosensors offer high selectivity owing to specific interactions, such as antigen-antibody interactions, thus demonstrating versatility and utility in molecular and cellular analyses and in selective ligand binding by aptamers [11]. However, antibody-based detection sensors have several limitations challenging in situ use, including the high cost of monoclonal ligands and their instability in harsh environments [12]. In contrast, aptamer-based detection sensors exhibit remarkable tolerance to extreme environmental conditions because of their relatively rigid nucleic acid backbones [13]. Nevertheless, isolating aptamers with high affinity and specificity is often costly, and all pathogenic bacteria may not be targetable by the isolated aptamers because of the diverse array of potential targets, reflecting the complexity of bacterial cell surfaces [14].

Recently, AMPs have been immobilized on interdigitated electrodes or magnetic nanoparticles to capture and detect pathogenic bacteria such as *Escherichia coli* [15], *Pseudomonas aeruginosa* [16], and *Listeria monocytogenes* [17] in biosensing systems [18,19]. AMPs offer several advantages as cost-effective components in biosensor development because of their broad-spectrum interactions with diverse pathogens, including bacteria, fungi, and viruses that possess lipoprotein envelopes [15]. The application of AMPs in biosensors relies on their ability to interact with bacterial cell-membrane components via hydrogen bonding, hydrophobic interactions, and electrostatic forces [20].

AMPs are highly stable under extreme environmental conditions and can semi-selectively bind to the cell surface of gram-negative species [21]. Certain AMPs have shown efficacy against pathogenic bacteria even under harsh autoclaving conditions and in the presence of chemical denaturants [22,23]. Additionally, AMPs provide a significant advantage as recognition elements for the detection of pathogenic bacteria because of their broad-spectrum activity [24]. Melittin, a cationic amphipathic molecule consisting of 26 amino acids, is known for its antibacterial activity and the ability to inhibit biofilm formation. Its structure includes polar and positively charged groups and hydrophobic regions, which facilitate interactions with negatively charged phospholipids in bacterial lipid bilayers [25,26]. Consequently, melittin exhibits exceptional broad-spectrum activity against both gram-positive and gram-negative bacteria [18].

The biorecognition layer in vertical-capacitance biosensors faces challenges related to the non-specific binding of small molecules in culture media. In blood culture media, non-specifically precipitated blood cells can interact with the recognition layer and form biofilms on the surface. To address this issue, vertical-capacitance sensors have been designed with a reduced gap between the glass substrate and gold electrode, thereby minimizing the recognition layer [7]. However, the total area of the recognition layer in the vertical-capacitance biosensor remains unchanged.

**Figure 1:**
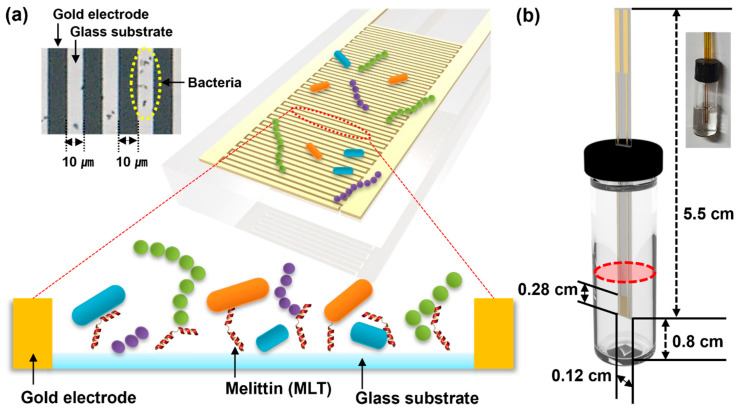
(**a**) Schematic of 10 μm wide gold electrodes with interdigitated with 10 μm glass substrate immobilized with melittin. The optical image shows bacteria captured on the glass substrate. (**b**) A vertical-capacitance sensor attached vertically to a 2 mL bottle. The inset shows a vertical-capacitance sensor.

In this study, the enhanced bacterial-capture capability of melittin was incorporated into the recognition layer of a vertical-capacitance biosensor. Biofilm formation poses a substantial challenge during bacterial detection in blood culture media using biosensors by creating a protective barrier on the surface [27,28,29]. Therefore, we hypothesized that a vertical-capacitance biosensor functionalized with melittin could effectively capture clinical bacteria. To test this hypothesis, we prepared a vertical-capacitance biosensor with melittin immobilized on the sensor surface between the electrodes (Figure 1). The melittin-immobilized vertical-capacitance sensor effectively captured both standard and pathogenic bacteria, as evidenced by real-time capacitance changes and bacterial growth measurements at different concentrations in the media. Additionally, the bacterial capture capabilities of the sensor were confirmed through real-time bacterial growth measurements in blood.

## 2. Methods

### 2.1. Reagents, Bacterial Cells, and Sensor Fabrication

3-(Aminopropyl)triethoxysilane (APTES), succinic anhydride (SA), *N*-hydroxysulfosuccinimide sodium salt (Sulfo-NHS), 1-(3-dimethyl-aminopropyl)-3-ethyl-carbodiimide (EDC), 2-(*N*-morpholino)ethanesulfonic acid solution (MES), and melittin were purchased from Sigma-Aldrich^®^ (St. Louis, MO, USA). The bacterial species used are presented in Table 1. Standard bacterial cell strains were obtained from the American Type Culture Collection (Manassas, VA, USA). Clinically isolated bacterial cells were provided by Yonsei University Severance Hospital (Seoul, Republic of Korea). Before measurement, all bacterial strains were sub-cultured on blood agar plates (KOMED Life Science Co., Ltd., Seoul, Republic of Korea) for 24 h. Mueller–Hinton broth (BD Biosciences, CA, USA) was used as the growth medium. For bacterial cell cultivation and growth, blood culture medium was prepared by mixing sheep blood (Synergy Innovation Co., Ltd., Gyeonggi-Do Republic of Korea) and distilled water with 0.8% bacto supplement (Difco & BBL). This study was approved by the Institutional Review Board of Severance Hospital (Yonsei University Health System; IRB No. 4-2017-1179).

A vertical-capacitance sensor array was fabricated on a glass substrate. Interdigitated Au electrodes of 10 µm width and 10 µm spacing were patterned using photolithography (Figure 1a), followed by the physical vapor deposition of a Cr/Au layer (5 nm/50 nm) and lift-off techniques. For bacterial culture, the lid of 2 ml vial was attached to the array sensor and sealed using epoxy. Before bacterial inoculation, the vertical-capacitance sensor array was cleaned with 70% ethyl alcohol.

For melittin immobilization on the surface of the biosensor, self-assembled monolayer carboxylated sensors were bioconjugated with melittin as described before, with suitable modifications [7,8,11]. Melittin was covalently immobilized on the capacitance biosensor surface by carbodiimide/*N*-hydroxysuccinimide coupling. Briefly, the prepared self-assembled monolayer carboxylated sensors were treated with a series of 0.4 mM APTES and 0.4 mM SA in ethanol for 12 h to activate carboxylic groups. Subsequently, the capacitance sensors were washed several times with distilled water, to which a solution of melittin (100 μL, 1 mg/mL) with 0.3 mM EDC and 0.3 mM Sulfo-NHS in 1 M MES was added and incubated for 24 h at room temperature. The uncoupled melittin was washed thoroughly with distilled water.

### 2.2. Data Collection

To induce bacterial growth, the bacterial solution (1 mL each of 10^0^, 10^1^, 10^2^, and 10^3^ CFU/mL) was added to the 2 mL vials in the vertical-capacitance sensor array containing blood culture solution (sheep blood:growth medium = 1:4, Figure 1b). The array was subsequently mounted onto an impedance analyzer (Cantis Co., Seoul, Republic of Korea) (Figure S1), which could simultaneously measure the capacitances of 16 sensors. The capacitance, impedance, and conductance were assessed using an LCR meter (Agilent 4284A, Santa Clara, CA, USA) with a peak-to-peak alternating current (AC) signal of 10 mV, across a frequency range from 0.5 to 200 kHz. The capacitance sensors were maintained inside an incubator, and the LCR meter was placed outside the incubator; they were connected via electrical connectors mounted on the incubator side and maintained at 37 °C. The capacitance, impedance, and conductance were measured simultaneously across the sensors using a data acquisition/switching unit (Agilent 34970A) connected to the LCR meter. The data were collected from each sensor every 990 s.

## 3. Results and Discussion

### 3.1. Vertical-Capacitance Sensor Characteristics

**Figure 2:**
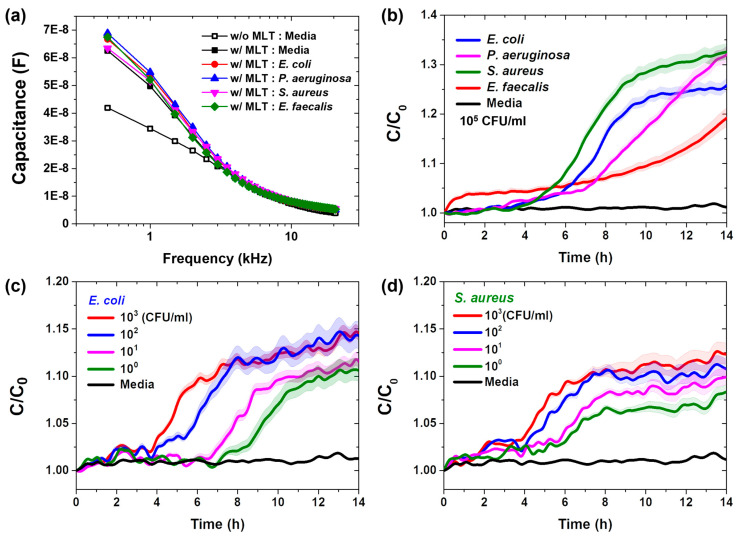
(**a**) Frequency−dependent capacitance measurements of the vertical−capacitance sensor in bacteria−free medium. Hollow black squares represent the values before melittin immobilization and solid black squares represent those after immobilization. The color symbols indicate the data collected from the melittin-immobilized vertical−capacitance sensor and 1 × 10^5^ CFU/mL bacterial solutions. Black symbols indicate the data collected from bacteria−free medium in the sensor immobilized with melittin. Red circles, *E. coli*; blue upward−pointing triangle: *P. aeruginosa*; Magenta downward−pointing triangle: *S. aureus*; Green diamond: *E. faecalis*. (**b**) Real−time capacitance measured for the melittin-immobilized capacitance array cultured with 1 × 10^5^ CFU/mL of different bacterial species. (**c**) Real−time capacitance measured for the melittin−immobilized capacitance array during the cultivation of *E. coli* at varying concentrations. (**d**) Real-time capacitance measured for the melittin−immobilized capacitance array during the cultivation of *S. aureus* at varying concentrations. The data are presented as the mean ± standard deviation, with a sample size of *n* ≥ 3.

We evaluated the frequency dependence of the capacitance before and after the immobilization of melittin onto the surface of the vertical-capacitance sensor in bacteria-free culture medium and in standard bacterial solutions applied to the biosensor surface following melittin immobilization. Consistent with the findings of previous studies, the immobilization of melittin on the sensor surface increased the capacitance (Figure 2a). The capacitance decreased as the frequency increased. These results suggest that melittin was successfully immobilized on the sensor surface, enabling the recognition of bacteria through its interaction with bacterial cell surfaces. The binding of both melittin and bacteria to the sensor enhanced its overall capacitance.

During bacterial growth, the changes in capacitance over time were monitored at a frequency of 0.5 kHz. The difference in the capacitance values between the bacteria-free medium and bacterial solutions increased following the addition of various bacterial solutions in the melittin-immobilized vertical-capacitance sensor at a concentration of 10^3^ CFU/mL (Figure S2) and 10⁵ CFU/mL (Figure 2b), which is the concentration frequently used for bacterial detection [30,31]. In this context, C₀ represents the initial capacitance value. At the beginning of the measurements, the capacitance remained almost constant. However, it increased rapidly after approximately 4 h and then stabilized. The normalized capacitance (C/C₀) curves, corresponding to the characteristic three phases of bacterial growth, were clearly observed. The lag and exponential phases of the bacterial growth curves were distinct, whereas the start of the stationary phase was dependent on the bacterial species. At this stage, the C/C₀ value indicated that most of the melittin on the sensor surface was bound to bacteria. Once the sensor surface was fully occupied by bacteria, the capacitance was expected to remain relatively constant despite additional bacterial growth.

For practical applications, bacterial detection should achieve limits ranging from 10 to 10^3^ CFU/mL [32,33]. To explore the relationship between real-time capacitance and bacterial concentration, measurements were conducted using melittin-immobilized sensors with *E. coli* and *S. aureus* at concentrations ranging from 10 to 10^3^ CFU/mL. Figure 2c shows that higher bacterial concentrations yielded a more rapid increase in C/C₀ values during the early stages of measurement and overall higher C/C₀ values. When the sensor surface was fully occupied by *E. coli*, the capacitance stabilized despite the continued bacterial growth. Additionally, the real-time capacitance data collected at a concentration of 10 CFU/mL were distinguishable from those of the bacteria-free medium. These findings suggest that the melittin-immobilized capacitance sensor is capable of detecting very low bacterial concentrations (potentially less than 10 CFU/mL).

Similar measurements were performed with *S. aureus* (Figure 2d). Capacitance increased more slowly for *S. aureus* than for *E. coli*, likely because of its smaller size and lower affinity for melittin. Although the C/C₀ ratio gradually increased during the stationary phase, real-time capacitance measured at a concentration of 10 CFU/mL was distinguishable from that of the bacteria-free medium. These results indicate that the melittin-immobilized vertical-capacitance sensor is suitable for real-time monitoring of bacterial growth.

### 3.2. Real-Time Pathogenic Bacterial Detection in Media

**Figure 3:**
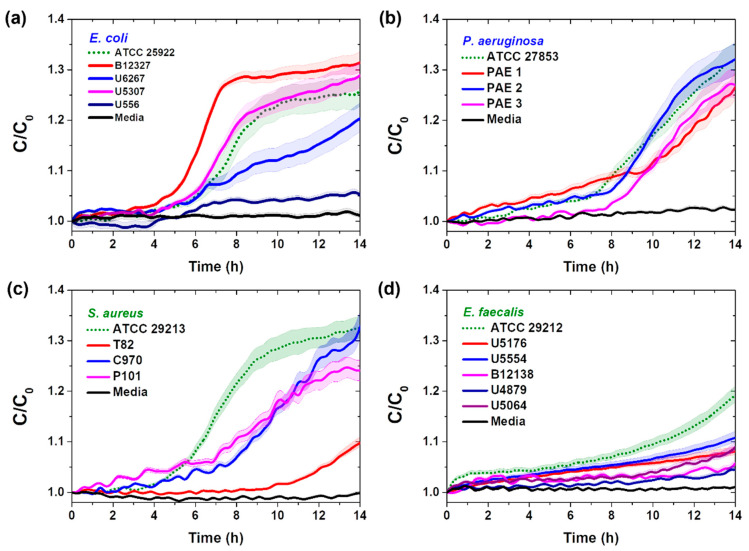
Real-time capacitance changes observed in a melittin-immobilized vertical-capacitance sensor treated with 1 × 10^3^ CFU/mL with standard bacteria (dot line) and 1 × 10^5^ CFU/mL of clinically isolated bacteria (solid line) of (**a**) *E. coli*, (**b**) *P. aeruginosa*, (**c**) *S. aureus*, and (**d**) *E. faecalis* in media at *f* = 0.5 kHz. The data are presented as the mean ± standard deviation, with a sample size of *n* ≥ 3.

Pathogenic bacteria of some species have complex cell surface structures. However, as reported by Terwilliger et al., melittin interacts with phospholipid molecules in the bilayer of both gram-positive and gram-negative bacterial cell membranes [28]. Therefore, we further investigated the selectivity of the melittin-immobilized vertical-capacitance sensor by conducting additional measurements using clinically isolated bacterial solutions of *E. coli*, *S. aureus*, *P. aeruginosa*, and *E. faecalis* at *f* = 0.5 kHz, as shown in Figure 3.

For the gram-negative bacteria *E. coli* and *P. aeruginosa*, the selectivity of the clinically isolated bacteria was similar to that of the standard bacteria at 4–8 h, except for *E. coli* U556 (Figure 3a,b). In contrast, for the clinically isolated gram-positive bacteria *S. aureus* and *E. faecalis*, selectivity was observed later than for standard bacteria (Figure 3c,d).

Our results align with those of Wilson et al., who demonstrated greater sensitivity of gram-negative bacteria (e.g., *E. coli*) to melittin than gram-positive bacteria (e.g., *S. aureus*) [28]. However, because of the high affinity of melittin, clinically isolated bacteria can be detected within 14 h through real-time capacitance changes. These results support the potential of the melittin-immobilized vertical-capacitance sensor for detecting pathogenic bacteria, regardless of the species.

### 3.3. Real-Time Detection of Pathogenic Bacteria in Blood

**Figure 4:**
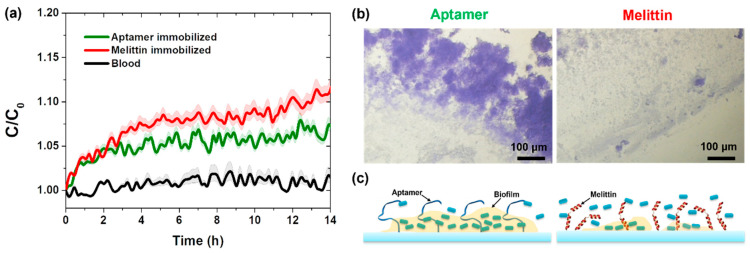
(**a**) Real-time capacitance changes measured for the aptamer or melittin-immobilized vertical-capacitance sensor treated with 1 × 10^3^ CFU/mL standard *E. coli*. The data are presented as the mean ± standard deviation, with a sample size of *n* ≥ 3. (**b**) Images of biofilm formation on aptamer- or melittin-immobilized sensors stained with crystal violet after 18 h. (**c**) Schematic of biofilm formation on aptamer- or melittin-immobilized sensors.

We prepared an aptamer-immobilized vertical-capacitance sensor using previously reported methods [7,8] and measured real-time capacitance changes in both aptamer- and melittin-immobilized capacitance sensors using a 10^3^ CFU/mL *E. coli* ATCC 25922 solution in blood culture media at a frequency of 0.5 kHz. As shown in Figure 4a, no capacitance difference was observed during the initial period; however, the sensitivity of the melittin-immobilized sensor became higher than that of the aptamer-immobilized sensor over time. Biofilm formation on both aptamer- and melittin-immobilized capacitance sensors was demonstrated using a staining method.

Less biofilm formation was observed on the melittin-immobilized capacitance sensor than on the aptamer-immobilized capacitance sensor (Figure 4b). Based on these results, we considered that bacterial detection in blood culture media using the melittin-immobilized vertical-capacitance sensor might be advantageous because of the enhanced bacterial mobility in blood culture media and reduced interference from biofilm formation (Figure 4c).

**Figure 5:**
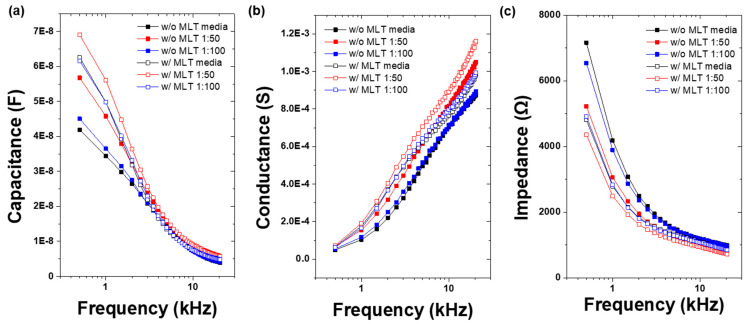
Frequency−dependence behavior of (**a**) capacitance, (**b**) conductance, and (**c**) impedance in bacteria−free medium and blood culture media of different blood:broth ratios (1:50 and 1:100) before and after immobilizing melittin on the vertical−capacitance sensor surface.

Before incubating clinically isolated bacteria in blood culture media, we assessed the frequency dependence of capacitance, impedance, and conductance in bacteria-free culture medium and blood culture media with different blood-to-broth ratios (1:50 and 1:100), both before and after melittin immobilization on the vertical sensor surface (Figure 5). Consistent with the findings of previous studies, we observed that as frequency increased, capacitance and impedance decreased whereas conductance increased [7,8,11]. Following melittin immobilization, both the capacitance and conductance increased in the bacteria-free medium and blood culture media, whereas the impedance decreased.

**Figure 6:**
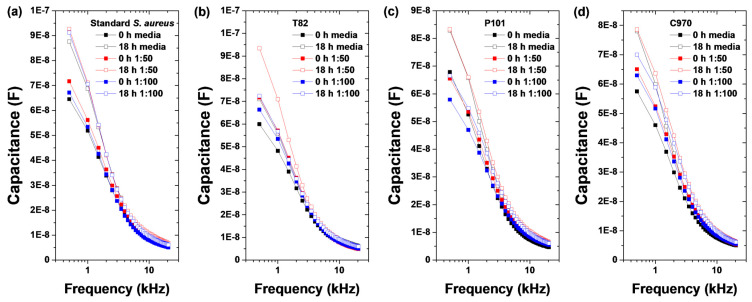
Frequency-dependence behavior of the capacitance in a melittin-immobilized vertical-capacitance sensor with (**a**) standard and (**b**–**d**) clinically isolated *S. aureus* strain (1 × 10^3^ CFU/mL) in culture media of different blood:broth ratios (1:50 and 1:100).

The standard procedure for culturing blood involved inoculating 5 mL of blood into 50 mL of broth, resulting in a blood-to-broth ratio of 1:10. Previous reports indicated that common pediatric pathogens can be recovered without delay from blood volumes as low as 0.5 mL cultured in media with blood-to-broth ratios of up to 1:100 [31]. We monitored the real-time capacitance at a frequency of 0.5 kHz while culturing clinically isolated *S. aureus* bacteria at a concentration of 10^3^ CFU/mL in blood culture media. Figure 6, Figures S3 and S4  show the frequency dependence of capacitance, impedance, and conductance of the melittin-immobilized vertical sensor at 0 and 18 h after culturing *S. aureus* in medium and blood culture media (blood-to-broth ratios of 1:50 and 1:100, respectively).

After 18 h of incubation, the capacitance and conductance increased, whereas the impedance decreased in both the medium and blood culture media (blood-to-broth ratios of 1:50 and 1:100). The capacitance, impedance, and conductance in the blood culture medium (1:50) exhibited large differences after 18 h of incubation at a low frequency. Additionally, significant differences in the capacitance, impedance, and conductance values were observed in the blood culture medium at a 1:100 ratio.

**Figure 7:**
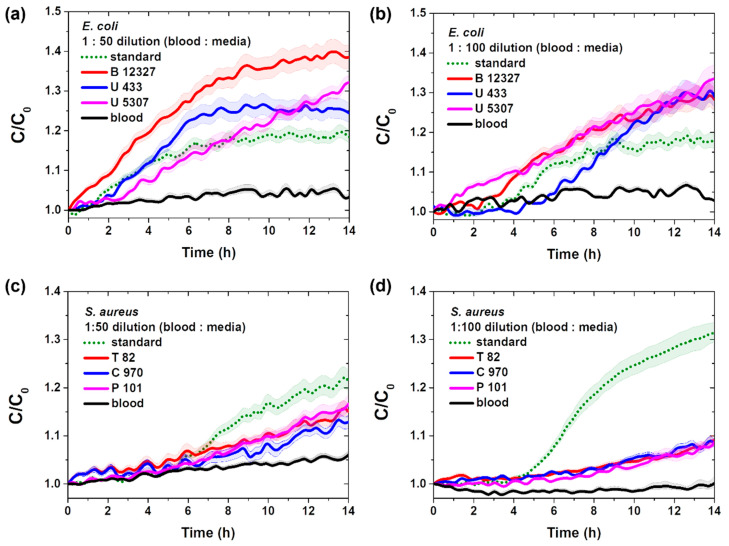
Real-time capacitance measured in a melittin-immobilized vertical-capacitance sensor with 1 × 10^3^ CFU/mL of *E. coli* or *S. aureus* in varying ratios of blood culture media (**a**,**c**) 1:50 and (**b**,**d**) 1:100. The real-time capacitance was measured at *f* = 0.5 kHz. The data are presented as the mean ± standard deviation, with a sample size of *n* ≥ 3.

Figure 7 illustrates the changes in real-time capacitance measured at a frequency of 0.5 kHz while culturing standard and clinically isolated strains of *E. coli* and *S. aureus* in blood culture media (blood-to-broth ratios of 1:50 and 1:100) at 10^3^ CFU/mL. The capacitance values changed more rapidly in the blood culture medium with a 1:50 ratio than in that with a 1:100 ratio, likely because of the higher availability of nutrients for bacterial growth. Additionally, Figure 7a,b demonstrates the capability of the vertical-capacitance biosensor immobilized with melittin to measure very small blood samples directly.

## 4. Conclusions

In this study, we demonstrate the effectiveness of the AMP melittin as a potent capture ligand for clinical bacteria in vertical-capacitance biosensors. We demonstrated melittin to be a powerful ligand for clinical bacteria in detection-sensing applications. They are not only well suited for detecting pathogenic bacteria in standard culture media but also enable bacterial detection in blood without any loss of activity during the process. In addition, considering the challenge posed by biofilm formation on the surface of bacterial biosensors, using a melittin-immobilized capacitance biosensor quickly and selectively detects the presence of bacteria with high sensitivity in blood. Therefore, a vertical-capacitance biosensor immobilized with melittin holds great promise for medical diagnostic applications aimed at preventing the spread of highly infectious diseases.

## Figures and Tables

**Table 1:** Bacterial strains used in the study.

Species of Bacteria	Standard Bacteria	Clinically Isolated Bacteria
	Gram-negative bacteria	
*E. coli*	ATCC 25922	B12327, U6267, U556, U5307
*P. aeruginoa*	ATCC 27853	PAE1, PAE2, PAE3
	Gram-positive bacteria	
*S. aureus*	ATCC 29213	T82, C970, P101
*E. faecalis*	ATCC 29212	U5176, U4879, U5064, B12138, U5554

## Data Availability

The original contributions presented in this study are included in the article/Supplementary Materials.

## Supplementary Materials

The following supporting information can be downloaded at: https://www.mdpi.com/article/10.3390/s25010012/s1, Figure S1: Photograph of the vertical-capacitance sensor measurement system. A 16-channel array was mounted on the incubator and maintained at 37 °C; Figure S2: Frequency-dependence behavior of capacitance in melittin-immobilized vertical-capacitance sensor with (a) standard and (b–d) clinical isolates of S. aureus (1 × 103 CFU/mL) in blood media with different blood:broth ratios (1:50 and 1:100). Figure S3: Frequency-dependence behavior of capacitance in melittin-immobilized vertical-capacitance sensor with (a) standard and (b–d) clinical isolates of S. aureus (1 × 103 CFU/mL) in blood media with different blood:broth ratios (1:50 and 1:100). Figure S4: Frequency-dependence behavior of impedance in melittin-immobilized vertical-capacitance sensor with (a) standard and (b–d) clinical isolates of *S. aureus* (1 × 10^3^ CFU/mL) in blood media with different blood:broth ratios (1:50 and 1:100).
