# Supplementary material for: Pathogenic Bacterial Detection Using Vertical-Capacitance Sensor Array Immobilized with the Antimicrobial Peptide Melittin
Source: Sensors (Basel). 2024 Dec 24;25(1):12. doi: 10.3390/s25010012 (PMC11722802; doi:10.3390/s25010012)
Supplement: Supplementary file 1 [file sensors-25-00012-s001.zip › sensors-3297701-supplementary.pdf]

Supplementary Data

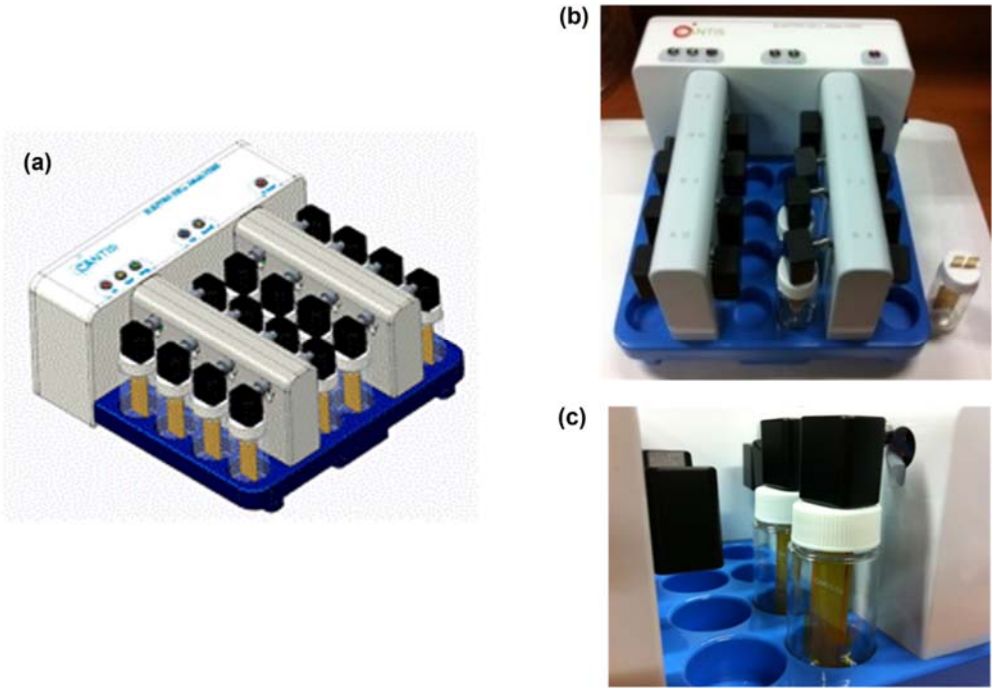

Figure S1. Photograph of the vertical-capacitance sensor measurement system. A 16-channel array was mounted on the incubator and maintained at 37 °C.

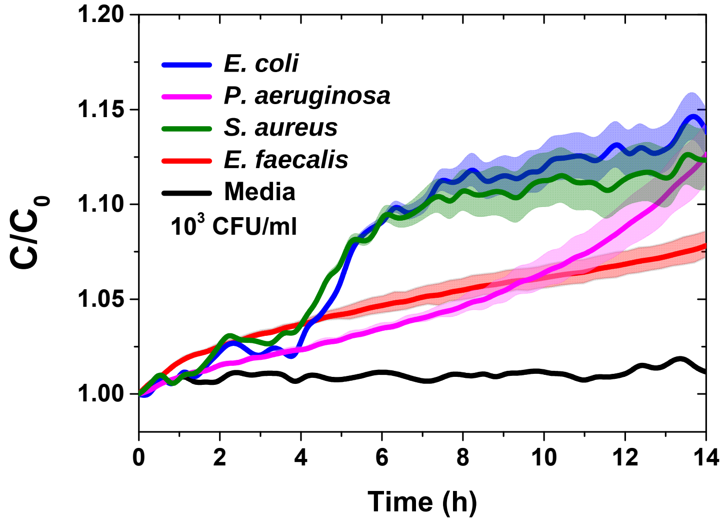

Figure S2. Real-time capacitance measured for the melittin-immobilized capacitance array cultured with 10^5 CFU/mL of different bacterial species.

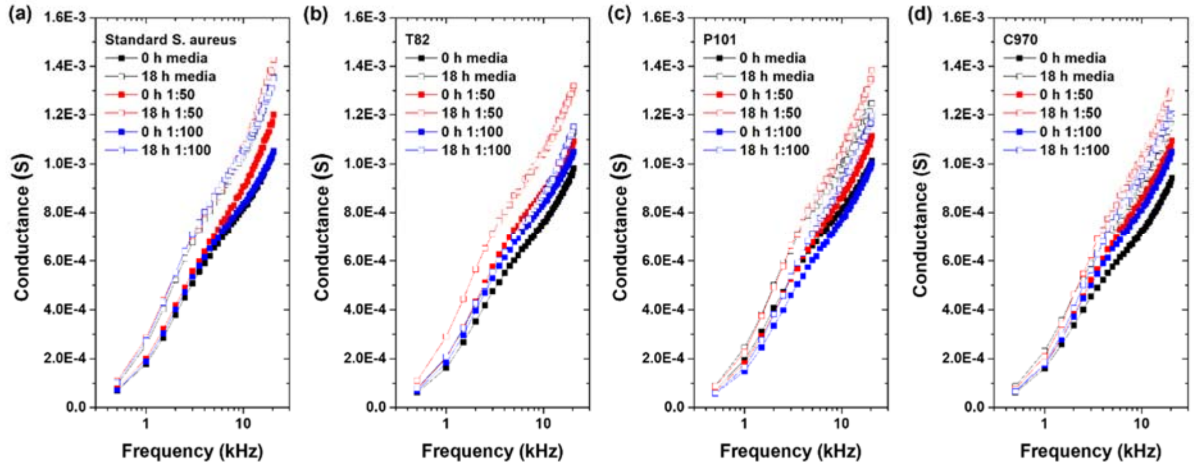

Figure S3. Frequency-dependence behavior of capacitance in melittin-immobilized vertical-capacitance sensor with (a) standard and (b) – (d) clinical isolates of *S. aureus* (10^3 CFU/mL) in blood media with different blood:broth ratios (1:50 and 1:100).

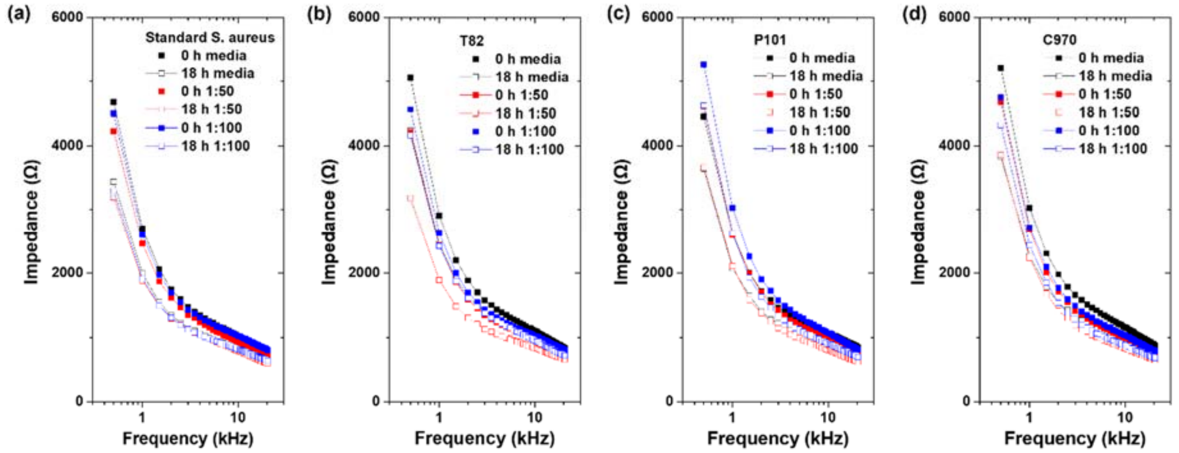

Figure S4. Frequency-dependence behavior of impedance in melittin-immobilized vertical-capacitance sensor with (a) standard and (b) – (d) clinical isolates of *S. aureus* (10^3 CFU/mL) in blood media with different blood:broth ratios (1:50 and 1:100).
